# How to help aortic dissection survivors with recovery?: A health promotion program based on the comprehensive theory of health behavior change and literature review

**DOI:** 10.1097/MD.0000000000033017

**Published:** 2023-02-17

**Authors:** Xiaorong Lang, Danni Feng, Sufang Huang, Yucheng Liu, Kexin Zhang, Xiaoxuan Shen, Jingjing Huang, Quan Wang

**Affiliations:** a School of Nursing, Tongji Medical College of Huazhong University of Science and Technology, Wuhan, Hubei Province, China; b Emergency Department, Tongji Hospital Affiliated to Tongji Medical College of Huazhong University of Science and Technology, Wuhan, Hubei Province, China.

**Keywords:** aortic dissection, health promotion, the Integrated Theory of Health Behavior Change

## Abstract

For aortic dissection survivors, health promotion can help them recover from the disease, which requires systematic program support. The aim of this study was to construct a health promotion program for aortic dissection survivors. Literature search, group discussion, and expert consultation were used. The Integrated Theory of Health Behavior Change was the theoretical basis of the program. Multiple medical-related databases were searched. Based on a literature search and group discussion, 3 primary items, 8 secondary items, and 34 tertiary items were formed. After 2 rounds of expert consultation (number of experts = 25), 3 primary items, 16 secondary items, and 54 tertiary items were retained. The authority coefficients of the 2 rounds of experts were 0.890 and 0.905, respectively. The Kendall *W* coefficient of the 2 rounds were 0.210 to 0.370 (*P* < .05) and 0.221 to 0.378 (*P* < .05), respectively. The mean importance value and coefficient of variation of each item were >3.5 and <0.25, respectively. The health promotion program constructed in this study was reasonable and scientific, which could provide a reference for clinical work.

## 1. Introduction

Aortic dissection (AD), an aneurysm caused by a tear in the tunica intima of a blood vessel leading to interstitial hemorrhage, and splitting (dissecting) of the vessel wall, has a high mortality rate with 14.3% to 29.3% in 30 days.^[1–3]^ With the development of diagnosis and treatment techniques, the survival rate of AD has been improved gradually.^[4]^ However, AD survivors still face many challenges, such as postoperative complications, impairment of physical function, decreased quality of life, and psychological trauma.^[5–8]^ Therefore, the health promotion of AD is one of the important means to help them recover from the disease.

Health promotion, which was defined as encouraging consumer behaviors most likely to optimize health potentials (physical and psychosocial) through health information, preventive programs, and access to medical care in MeSH terms, is being widely used to improve patient rehabilitation. It was reported that the health promotion program improved the health perception, perceived exercise benefits self-efficacy, exercise-related activity level, and treatment adherence of coronary artery patients.^[9,10]^ In addition, health promotion was considered a cost-effective means of reducing cardiovascular events and mortality.^[4]^ However, there are few health promotion programs for AD.

Health promotion requires people to initiate and maintain health behavior changes. The Integrated Theory of Health Behavior Change (ITHBC) views behavior change as a dynamic iterative process and purports health behavior change can be enhanced by fostering knowledge and beliefs, increasing self-regulation skills and abilities, and enhancing social facilitation.^[11]^ Therefore, we aim to construct a health promotion program for AD survivors based on ITHBC to help promote recovery. The plan is implemented by professional medical personnel, including cardiac rehabilitation specialist staff, cardiologists, nurses, rehabilitation (exercise) therapists, pharmacists, nutritionists, and psychotherapists, and requires the cooperation and participation of patients and relatives during discharge and follow-up.

## 2. Methods

### 2.1. Set up a research group

The study team consisted of 10 members, 1 chief physician, 1 chief nurse, 2 charge nurses, 1 doctoral student in nursing, and 5 master’s students in nursing. The chief physician and chief nurse practitioner were responsible for the discussion, modification of the health promotion program, and expert selection. The supervising nurse practitioner, doctoral and master’s students were responsible for reviewing relevant domestic and foreign literature, screening, initial development of the program, revision, preparation of expert correspondence questionnaires, collection, and organization of data, discussion, and analysis of results. The discussion of expert advice, responses, and inclusion and exclusion criteria of indicators was decided jointly by the group.

### 2.2. Constructing the preliminary draft of the health promotion program for AD survivors

Search PubMed, Embase, Web of Science, CINAHL, Medline, Up To Date, BMJ Best Practice, Cochrane Library, JBI, and other websites or databases using “aortic disease,” “health promotion,” “management,” “guideline,” and “consensus” as English search words. At the same time, we also consulted the American Heart Association, European Society of Thoracic Surgeons, European Association for Cardio-Thoracic Surgery, British Thoracic Society, American Thoracic Society, American Association for Thoracic Surgery, Japanese Association for Thoracic Surgery, Thoracic Surgeons Branch of Chinese Medical Doctor Association, Thoracic Society of Australia and New Zealand, Canadian Thoracic Society, and other disciplinary websites. The research team analyzed, summarized, and synthesized the contents consulted, and initially formed a health promotion plan for AD survivors that included 3 primary items, 8 secondary items, and 34 tertiary items, and designed the expert consultation questionnaire accordingly.

### 2.3. Designing expert consultation questionnaires

The expert consultation questionnaire was divided into 3 sections: Letter to experts: a brief description of the study’s background, purpose, significance, content, and expert correspondence method. Expert rating scale: 3 first-level items, 8 second-level items, and 36 third-level items were included. The experts were asked to judge the importance of each item based on their theoretical and practical experience using the Likert 5-point scale (very unimportant = 1 point, unimportant = 2 points, average = 3 points, important = 4 points, very important = 5 points). Set up the “Modify” column, “Delete” column, and “Add” column, and ask the experts to modify and add to the entries. The mean importance value ≤ 3.5, coefficient of variation (CV) > 0.25, and full score ratio ≤ 20% were used as exclusion criteria.^[12]^ Basic information questionnaire of experts including general information of experts, judgment basis, and familiarity rating of investigation content. The basis of judgment is divided into 4 items: theoretical analysis, practical experience, reference to domestic and foreign information, and intuitive judgment, and each item is divided into 3 levels: large, medium, and small according to the degree of influence on the expert’s judgment. The degree of familiarity was divided into very familiar, more familiar, average, less familiar, and unfamiliar, with values of 0.9, 0.7, 0.5, 0.3, and 0, respectively.^[13]^

### 2.4. Selecting experts

A purposive sampling method was used to select experts in cardiovascular surgery from 11 tertiary-level general hospitals in ten regions of Gansu, Guangzhou, Henan, Jiangsu, Shanxi, Shanghai, Sichuan, Hubei, Zhejiang, Hunan to conduct correspondence on the scale content. Selection criteria: 5 years or more in the medical care of cardiovascular diseases (CVDs); associate senior title or above; bachelor degree or above; able to cooperate to participate in the whole consultation of this study; and familiar with AD disease and highly motivated to this study, able to provide more comprehensive opinions and suggestions from different perspectives.

### 2.5. Implementation of expert consultation

From August to November 2022, a correspondence consultation was conducted with experts who met the inclusion criteria. In total, 2 rounds of expert consultation were issued, with 25 questionnaires issued in each round. Twenty-five experts were sent correspondence questionnaires by email in the first round, detailing the purpose and requirements of the study, and 25 valid questionnaires were returned; team members calculated data, summarized textual comments, discussed, and deleted, expanded, and modified some entries to form the second round of expert consultation questionnaires. In the second round, the questionnaire was sent to 25 experts who had completed the first round of the study, and 20 valid questionnaires were returned.

### 2.6. Statistical methods

SPSS 24.0 (IBM Corp., Armonk, NY) was used for the statistical analysis of the data. Normally distributed measurement data were described by mean ± standard deviation, and count data were described by frequency, composition ratio, and rate. Expert motivation was reflected by the questionnaire return rate, >70% was considered as effective consultation, and the authority of expert opinion was expressed by the authority coefficient (Cr), which was calculated by the coefficient of judgment basis (Ca) and the coefficient of familiarity (Cs), the calculation formula is: Cr = (Ca + Cs)/2.^[14]^ The degree of coordination of expert opinion was described by CV and Kendall *W* coefficient. Differences were considered statistically significant at 2-tailed *P* value < .05.

## 3. Results

### 3.1. General information of experts

After 2 rounds of expert consultation, 25 experts completed the expert consultation. Among them, 5 were male and 20 were female; age ranged from 37 to 58 (46.56 ± 5.21) years; working years ranged from 11 to 39 (25.12 ± 7.31) years; 5 had a doctoral degree, 7 had a master’s degree, and 13 had a bachelor’s degree; 9 had a senior title and 16 had an associate title; 13 had a professional field of clinical nursing, 7 had clinical management, and 5 had a clinical medicine.

### 3.2. Active level of experts

The effective return rate of the questionnaires represents the motivation of experts. Hundred percent and 80% of the questionnaires for the 2 rounds of expert consultation were effectively returned. Eighty-four percent and 20% of the experts made comments and suggestions for the 2 rounds of consultation, respectively, indicating that the experts were highly motivated.

### 3.3. Authority of expert opinion

In this study, the first round of expert consultation Cr = (0.828 + 0.976)/2 = 0.890, and the second round of expert consultation Cr = (0.830 + 0.980)/2 = 0.905.

### 3.4. Coordination degree of expert opinion

In the first round of expert consultation, the Kendall *W* coefficient of the primary, secondary, and tertiary items were 0.201, 0.302, and 0.370, respectively, *P* < .05; The Kendall *W* coefficient of the primary, secondary, and tertiary items of the second round of expert consultation were 0.221, 0.388, and 0.378 respectively, *P* < .05.

### 3.5. Results of expert consultation

The mean importance value and CV at each level in the 2 rounds of expert consultation were shown in Table 1. After the 2 rounds of expert consultation, the contents of the entries were revised after the panel discussion. The experts in this round did not propose any revision to the 3 primary items, but the secondary items were revised as follows: The experts thought that the specific contents listed in the secondary item of “health education” was difficult to cover all the individualized health education needs of AD, and suggested that the secondary item be simplified, which was adopted. The secondary item “health education” was simplified to include disease knowledge guidance, wound guidance, medication guidance, diet guidance, lifestyle guidance, activity guidance, review guidance, risk prevention guidance, emotional support, and other rehabilitation guidance. The experts thought that the secondary item of “self-management” was duplicated with the content under “health education,” so they suggested deleting this part, which was adopted. The tertiary items were revised as follows: The experts thought that “popularize the causes, risk factors and clinical symptoms of AD” was not well expressed, and suggest to change it to “introduced etiology, high risk factors, clinical features and hazards of AD to patients,” which was adopted. The experts thought that the expression of the item “prompt medical consultation is needed when there are symptoms such as fever, decreased skin temperature, the disappearance of dorsalis pedis artery pulsation, chest discomfort, chest pain, abdominal pain, low back pain, and bleeding tendency in those taking oral anticoagulants” was inaccurate, as there were many uncomfortable symptoms after AD, which cannot be accurately summarized. It was suggested to amend it to “inform patients to seek medical advice in time if discomfort symptoms occur after discharge,” which was adopted. Experts said that “for middle-aged and elderly patients, diet should be equipped with a reasonable diet” was repetitive, so they suggested deleting it, which was adopted. Experts said that the expressions “replacement” and “decubitus ulcer” were not standardized, and suggested replacing them with “use” and “pressure injury,” which was adopted. The expert suggested that the item under “Enhancement of beliefs” could be added by referring to the “Health Belief Model,” which was adopted. The expert suggested that “guiding patients to develop individualized rehabilitation plans” should be changed to “developing individualized rehabilitation plans through patient-centered consultation,” which was adopted. The expert suggested verifying the blood pressure control target and weight control target and suggested adding the blood glucose control target, reasonable diet target, and medication compliance plan, these items were modified and supplemented after group discussion. The experts suggested that overweight and obesity should be expressed by BMI index instead, which was adopted. The experts suggested that imaging follow-up should also be an important part of self-monitoring, so they suggested adding this item, which was accepted. The expert suggested regular review of blood lipids and emphasized the time and index of regular review, which was adopted. The expert suggested that the content under “Self-assessment” should be expanded to include the appropriate response measures after the assessment, which was adopted. The expert suggested to change “encourage family members/caregivers to face the disease correctly, avoid aggravating the patient’s guilt due to financial problems, obtain the cooperation and support of family members/caregivers, and create a happy and harmonious family atmosphere” to “encourage family members/caregivers to face the disease correctly, create a relaxed and happy living environment, and promote the patients physical and mental recovery,” which was adopted. The expert suggested replacing “listen to patients’ worries and provide guidance on techniques to cope with negative emotions” with “provide psychological counseling and support channels, listen to patients’ worries, provide guidance on techniques to cope with negative emotions, and promote patients’ positive coping with negative emotions,” which was adopted. The expert suggested adding “a video on popular science education for patients with AD.” The expert suggested adding the item “disseminate medical policies and measures related to CVD treatment to provide social support,” which was adopted after discussion by the group members. The expert suggested to add the item “build a health promotion management platform for patients with AD by relying on the smart medical module of the hospital’s WeChat public number to facilitate communication between medical staff and patients and give health guidance,” which was adopted after discussion by the group members. The final promotion program for AD contained 3 primary items, 16 secondary items, and 54 tertiary items, as shown in Table 2.

**Table 1:** The mean importance value and coefficient of variation of indicators at all levels in 2 rounds of expert consultation.

Round	Index grade	Mean of importance value	CV
First round (n = 25)	Level I items	3.84–4.64	0.10–0.17
	Level II items	3.88–5.00	0–0.18
	Level III items	3.64–5.00	0–0.23
Second round (n = 20)	Level I items	4.65–5.00	0–0.13
	Level II items	4.20–5.00	0–0.15
	Level III items	3.95–5.00	0–0.21

CV = coefficient of variation.

**Table 2:** Health promotion program for patients with aortic dissection.

A1 Knowledge belief	A1-1 disease knowledge guidance	A1-1-1 Introduced etiology, high-risk factors, clinical features, and hazards of AD to patients.
		A1-1-2 Inform patients of the assessment, development, impact, and management of common postoperative complications.
	A1-2 wound care instructions	A1-2-1 Instruct patients on proper wound management and keep the wound clean and ventilated until it heals.
		A1-2-2 Instruct patients to check the incision regularly for signs of infection. If there is redness, swelling, distension, exudation, or foreign body in the incision, consult the doctor and go to the outpatient department for review.
	A1-3 medication guide	A1-3-1 Popularize the importance of timely and rational drug use, so as to reduce the incidence of missed or excessive drug use.
		A1-3-2 Guide the observation and coping skills of adverse reactions during the use of drugs to control blood pressure, heart rate, and anticoagulants.
	A1-4 diet guidance	A1-4-1 Guide the patient to follow a light diet, easy to digest, low cholesterol, low fat, low salt diet. Eat more soy products, milk, high-quality protein, and other foods. Do not eat animal offal and overnight meals, avoid oil-fried, spicy food.
		A1-4-2 Inform patients of the importance of maintaining a smooth stool, guiding a reasonable diet, eat more fruits and vegetables, grains, and other foods rich in dietary fiber.
		A1-4-3 Have more meals a d but less food at each and avoid overeating.
		A1-4-4 Tell patients to cut down on caffeinated beverages, such as coffee and green tea.
	A1-5 guide to living habits	A1-5-1 Inform patients of the importance of quitting smoking and drinking and provide strategies.
		A1-5-2 Actively control blood lipid, blood glucose, blood pressure, and heart rate.
		A1-5-3 For patients with stool secret history in the past can be routinely equipped with some laxative drugs at home, such as dumic, glycerine enema, etc.
		A1-5-4 Avoid a sudden increase in intra-abdominal pressure, do not hold your breath, cough, sneeze, or force defecation.
		A1-5-5 Explain to patients the harm of staying up late, tiredness, emotional tension, and excessive anxiety, guide patients to develop a regular schedule of early to bed and early to rise, reduce daytime sleep time, improve the quality of sleep at night, and avoid adverse stimuli.
	A1-6 activities guidance	A1-6-1 After discharge, patients should be guided to rest, and the correct way to get out of bed (30 s: sitting on the bed for 30 s → standing on the bed for 30 s → walking on the bed → walking in the room → walking in the corridor gradually) should be informed, so as to avoid sudden changes in posture, especially at night. Always have someone with him/her when he/she gets out of bed.
		A1-6-2 Advise patients not to exercise vigorously within 3 mo after surgery, such as running, heavy physical labor, long driving, and pay attention to the combination of work and rest.
		A1-6-3 For patients after thoracotomy, do not pull the chest when getting up in bed within 3 mo to avoid affecting the sternal healing.
		A1-6-4 After the physical condition is gradually improved (usually 3 mo after surgery, patients can have normal activities), it is recommended to gradually increase the amount of exercise, and prohibit sports such as competitive sports, heavy manual labor, lifting heavy objects, and twisting the body.
		A1-6-5 Avoid sedentary, exercise and meals more than an hour apart, not early morning exercise.
	A1-7 review guidance	A1-7-1 Instruct patients to review at 1 mo, 3 mo, 6 mo, 1 year after discharge, and annually thereafter.
		A1-7-2 Inform patients to seek medical advice in time if discomfort symptoms occur after discharge.
	A1-8 risk prevention guidance	A1-8-1 Instruct patients to pay attention to the weather changes, increase or decrease clothing in time, and prevent colds.
		A1-8-2 Advise patients to avoid long-term exposure to areas with severe air pollution and to avoid crowded places.
		A1-8-3 Inform patients of the harm of falling, reduce the risk factors of falling in the environment and explain the preventive measures and self-rescue methods.
	A1-9 emotional support	A1-9-1 Encourage patients to take an active part in social activities.
		A1-9-2 Inform patients of coping strategies that deflect negative emotions (e.g. hobbies, social engagement).
	A1-10 other rehabilitation-related guidance	A1-10-1 Instruct patients and their family members/caregivers to cough and discharge sputum correctly and turn over regularly to avoid lung infection.
		A1-10-2 Guide paraplegic patients and their families to adopt correct rehabilitation training methods, such as replacing soft and comfortable mattresses, turning over, massaging, scrubbing, and cleaning frequently, so as to avoid the occurrence of pressure damage.
	A1-11 strengthen belief	A1-11-1 Inform patients of the potential threat and seriousness of noncompliance with health behaviors.
		A1-11-2 Inform patients of the importance and benefits of health promotion after discharge.
		A1-11-3 Understand the difficulties and barriers patients may face in adopting healthy behaviors.
		A1-11-4 Affirm the improvement of patients’ health behaviors and disease indicators to improve the enthusiasm of patients to participate in health promotion programs.
		A1-11-5 Prompt and guide patients when they may be facing factors contributing to unhealthy behaviors.
B1 self-regulation	B1-1 goal setting	B1-1-1 Take patients as the main body to jointly discuss and formulate individualized rehabilitation plans.
		B1-1-2 Instructed patients to perform 150 min of light to moderate aerobic exercise (3–5 METs) per wk for 30 min each time after stabilization to reduce resting blood pressure and enhance cardiopulmonary function.
		B1-1-3 Inform patients that the optimal target for blood pressure control after discharge is <120/80 mm Hg with a heart rate of 60–80 beats/min. For the elderly or those with comorbidities, the adjustment is made according to the lowest level tolerated by the patient.
		B1-1-4 In AD patients with diabetes mellitus, fasting blood glucose is generally controlled at 5.0–7.0 mmol/L, non-fasting blood glucose is <10.0 mmol/L, and glycated hemoglobin target was <7.0%. For diabetic patients with a short course of disease, long life expectancy and no obvious cardiovascular disease, the target glycosylated hemoglobin is <6.5%. For patients with a long course of disease, a history of severe hypoglycemia, a short life expectancy, significant microvascular or macrovascular complications, or severe comorbidities, the glycemic control target can be appropriately relaxed. The target glycated hemoglobin should be controlled at 7.5%–8.0%, but acute hyperglycemia symptoms or related complications should be avoided.
		B1-1-5 Increase the intake of vegetables and fruits, coarse grains, legumes, and fish; Reduce fat meat, and pickles intake; Salt intake < 6 g; Edible oil intake < 25g/d; Quit smoking wine; The cooking methods of frying and frying and roasting are changed to steaming, boiling and braising.
		B1-1-6 Individuals who are overweight (BMI ≥ 24.0 kg/m^2^), obese (BMI ≥ 28.0 kg/m^2^), and at risk for cardiovascular disease should receive a target weight loss of 5%–10% within 3–6 mo
		B1-1-7 Inform patients of the time and dosage of the medication and help the patient take corresponding measures (such as setting an alarm clock, family member/caregiver reminding) to prevent mistaking.
	B1-2 self-monitoring	B1-2-1 Instruct patients to measure blood pressure, heart rate, pulse, blood glucose, and weight correctly, inform the matters needing attention during measurement, and record in real-time.
		B1-2-2 Instruct patients to check blood lipids regularly. Liver function, creatine kinase, and blood lipids were monitored every 4–6 wk for initial medication or adjustment of lipid-lowering therapy, and the safety and efficacy were initially evaluated and the compliance of patients was understood. If the patients were safe and tolerated, the patients were reexamined after 3 mo, and repeated monitoring was performed until safety was reached, and then the patients were reexamined every 6–12 mo.
		B1-2-3 Patients should be informed that imaging follow-up should be performed on time before discharge, 3, 6, and 12 mo after surgery, and every year thereafter, and the follow-up frequency of special patients should be determined according to the doctor’s recommendation.
	B1-3 self-evaluation	B1-3-1 Guide patients to assess their own cardiovascular risk, including family history of early onset of cardiovascular disease, familial hypercholesterolemia, and other cardiovascular disease risk factors, such as smoking, hypertension, diabetes, blood lipid levels, obesity, or increase risk of cardiovascular disease (CVD) comorbidities, inform the medical staff, adopt corresponding treatment measures to reduce disease risk.
		B1-3-2 Instruct patients to evaluate their frailty state according to the scale, and take corresponding measures (such as balanced nutrition, micronutrient supplementation, exercise training, social activities, etc.) or seek medical help in time.
		B1-3-3 Guide patients to assess their own stress and stressors, and timely self-regulation or seek professional help to deal with it.
	C1-1 family/caregiver support	C1-1-1 Invite family members/caregivers to participate in the monitoring process of health promotion to improve patients’ compliance.
		C1-1-2 Encourage family members/caregivers to communicate with patients about their feelings about health promotion to timely understand patients’ psychological and physical changes, provide emotional support, seek help in time, and solve problems.
		C1-1-3 Encourage family members/caregivers to correctly face the disease, create a relaxed and pleasant living environment, and promote the physical and mental recovery of patients.
C1 social facilitation	C1-2 medical staff support	C1-2-1 Provide psychological counseling and support channels, listen to patients’ troubles, guide patients to cope with negative emotions skills, and promote patients to actively cope with negative emotions.
		C1-2-2 Establish a WeChat group for peer support of aortic dissection, and an online multidisciplinary team for aortic dissection health promotion to provide patients with science popularization and education videos and disease-related support and guidance.
		C1-2-3 An informed discussion of CVD risks and treatment benefits, based on the patient’s needs. To disseminate medical policies and measures related to the treatment of cardiovascular diseases and provide social support.
		C1-2-4 Relying on the smart medical module of the hospital’s WeChat public account, build a health promotion and management platform for patients with AD to promote communication between patients and medical staff and give health guidance.

AD = aortic dissection, CVD = cardiovascular disease.

## 4. Discussion

Continuous health promotion after treatment is an important issue for AD survivors. We constructed a health promotion program based on ITHBC and formed the specific content of the program from three aspects: knowledge and beliefs, self-regulation skills and abilities, and social facilitation. All entries in the health program were derived from search results in professional databases.

Knowledge and beliefs include health education and belief enhancement for AD survivors. Health education is based on condition-specific factual information, such as etiology and symptoms of diseases, medication knowledge, improvement of living habits, exercise intensity, and risk prevention. Karen et al pointed out that integrating disease-related information in patient education can improve deeper knowledge and enhance patients’ perception of cardiac rehabilitation.^[15]^ Poor living habits, such as smoking, drinking, and obesity, are often potential risk factors for AD.^[16–18]^ The implementation of heart health education was helpful to improve patients’ adherence to a healthy lifestyle.^[19]^ Exercise rehabilitation has always been a topic of caution in AD. AD survivors may not be aware of appropriate exercises to help recovery.^[20]^ Therefore, exercise-related health education is very necessary. Belief enhancement aimed to improve personal perceptions about the specific health condition or health behavior, which can correct the progression of health behaviors in patients with CVD.^[21]^ In our program, the enhancement of beliefs for AD survivors was mainly achieved by emphasizing the potential threat and seriousness of unhealthy behaviors and the importance and benefits of health promotion. At the same time, the self-efficacy of the participants was improved by affirming them. Because perceived benefits were one of the important predictors of protective health behavior,^[22]^ and self-efficacy affected the choice of behavior and the amount of effort and perseverance that goes into it.^[23]^ The timely attention and resolution of obstacles were also the support of beliefs.

Self-regulation has important implications for individual trajectories of well-being.^[24]^ In our health promotion program, self-regulation consists of 3 parts: goal setting, self-monitoring, self-evaluation, and self-management. Goal setting is an effective behavior change technique that was considered a fundamental component of successful interventions.^[25]^ We set targets for exercise, blood pressure, blood glucose and lipids, weight, and diet for AD survivors, and considered self-monitoring as an important means to achieve the goals. Self-evaluation was designed to enable AD survivors to master changes in their disease and identify potential risks since it was often they, not others, who feel something wrong first. The self-regulation of AD survivors involved many aspects, such as disease, drugs, emotions, and living habits. Health promotion for AD survivors should be systematic, so self-management is not only focused on the condition. We hope that the self-regulation of AD survivors can enhance their sense of participation in disease management and improve the effectiveness of health promotion.

Social facilitation was focused on social support from family, caregivers, and medical staff. In the context of CVD, studies showed clear associations between social support and health outcomes. Various studies have shown that CVD was associated with a poorer prognosis in patients with low social support.^[26–28]^ We hoped to provide emotional support and access to medical resources for AD survivors through social support, and play a role in urging the change of disease and self-management.

In addition to ensuring the reasonableness of contents, the health promotion program was scientific and reliable. The 25 experts who participated in this study were all from large tertiary hospitals. Their professional fields involved clinical nursing, clinical management, and clinical medicine, and they were familiar with AD. It can be seen that the selected experts have rich professional knowledge and practical experience in this field, and are representative of the discipline to a certain extent. At the same time, >80% of the responses to the consultation questionnaire and most of the suggestions for content modification represent the experts’ support for this study. The authority coefficient of the experts in this study was 0.890 and 0.905, which was highly authoritative. After 2 rounds of consultation in this study, the mean importance value of each item was all >3.5, the CV of each item was all <0.25, and the coordination coefficient of expert opinions in the 2 rounds was statistically significant (*P* < .05), indicating that the expert opinions tended to be consistent.

To the best of our knowledge, this was the first systematic health promotion program developed to assist AD survivors in their recovery from the disease. At the same time, our program came from a wide range of literature, after focus group discussion and expert consultation, and the content was credible.

However, there were still some limitations. First, this health promotion program has not been used in clinical practice yet. In the subsequent study, we will apply it to clinical work and continue to test the feasibility of the program. Secondly, despite the consensus of expert opinion after 2 rounds of consultation, due to the limited scope of expert consultation and language limitations of literature retrieval, the coverage of the program may not be comprehensive enough. And clinical application may not be completely consistent with expert opinion. Therefore, we will continue to optimize the protocol during clinical application. Finally, how to ensure compliance with the participation of patients and their relatives and how to evaluate the effects of health promotion programs need to be further explored.

## 5. Conclusion

This study constructed a health promotion program for AD survivors through an extensive literature review, expert panel formation, and expert consultation. The method adopted in this study was scientific and reasonable, and the process was rigorous and reliable, which could provide a reference for the health promotion of AD survivors.

## Acknowledgments

Thanks to all authors for their contributions.

## Author contributions

**Conceptualization:** Xiaorong Lang, Danni Feng.

Data curation: Danni Feng.

Funding acquisition: Sufang Huang.

Investigation: Xiaorong Lang, Danni Feng, Yucheng Liu, Kexin Zhang, Xiaoxuan Shen, Jingjing Huang, Quan Wang.

Methodology: Xiaorong Lang.

Resources: Sufang Huang.

Software: Xiaorong Lang, Danni Feng.

Supervision: Sufang Huang.

Writing – original draft: Xiaorong Lang, Danni Feng.

Writing – review & editing: Xiaorong Lang, Danni Feng.
